# Hemoptysis as primary manifestation in three women with choriocarcinoma with pulmonary metastasis: a case series

**DOI:** 10.1186/s13256-017-1256-9

**Published:** 2017-04-16

**Authors:** Wenping Zhang, Bao Liu, Jizhen Wu, Beibei Sun

**Affiliations:** Department of Respiratory and Critical Care Medicine, People’s Hospital of Henan Province, Zhengzhou University, Zhengzhou, Henan Province China

**Keywords:** Hemoptysis, Gestational choriocarcinoma, hCG, Pulmonary metastases

## Abstract

**Background:**

Gestational choriocarcinoma is the most common gestational trophoblastic neoplasia; it is often secondary to hydatidiform mole, as well as to abortion, ectopic pregnancy, premature delivery, or term delivery. Approximately 60% of patients with choriocarcinoma develop pulmonary metastases, but for patients with a respiratory condition, choriocarcinoma with lung metastasis is a relatively rare lung cancer diagnosis. Three cases of choriocarcinoma with pulmonary metastasis who had the primary symptom of hemoptysis are described.

**Case presentation:**

This case report describes a 35-year-old Chinese woman of Han nationality, a 23-year-old Chinese woman of Han nationality, and a 46-year-old Chinese woman of Han nationality whose primary symptom was hemoptysis and different chest imaging manifestations; they were finally diagnosed as having pulmonary metastatic choriocarcinoma. All patients had low risk factors, including abortion, hydatidiform mole, and ectopic pregnancy. Human chorionic gonadotropin played an important role in choriocarcinoma diagnosis.

**Conclusions:**

Based on the diagnosis and treatment of the three patients, we suggested that for women with pregnancy history and hemoptysis (particularly in the presence of risk factors such as abortion, hydatidiform mole, ectopic pregnancy, and >35-years old), choriocarcinoma may be the possible diagnosis or at least the main differential diagnosis.

## Background

Gestational choriocarcinoma is the most common gestational trophoblastic neoplasia; it is often secondary to a hydatidiform mole and to abortion, ectopic pregnancy, premature delivery, or term delivery. This malignant tumor grows rapidly and metastasizes in the lungs, brain, liver, kidneys, intestine, pelvis, and vagina. Approximately 60% of patients with choriocarcinoma develop pulmonary metastases. However, for patients with a respiratory condition, choriocarcinoma with lung metastasis is a relatively rare lung cancer diagnosis. Three cases of choriocarcinoma with pulmonary metastasis who had the primary symptom of hemoptysis are described.

## Case presentation

### Case 1

**Fig. 1:**
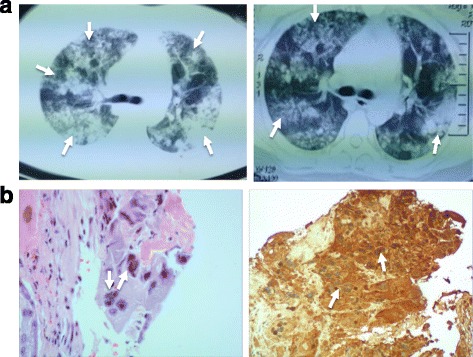
**a** Chest computed tomography at presentation shows diffused patchy infiltrates (*arrows*) throughout both lungs. **b** (Left) Microscopically atypical cell clusters (*arrows*) are found in the lung tissue, and (right) human chorionic gonadotropin (*arrows*) are detected via immunohistochemistry (hematoxylin and eosin × 200)

A 35-year-old Chinese woman of Han nationality presented with hemoptysis of 16 days’ duration. She had shortness of breath during physical exertion, cough, and sputum with blood when she was 36 weeks pregnant. She gave birth to a healthy baby boy via caesarean section 10 days prior. A computed tomography (CT) scan of her chest revealed a diffuse, patchy high-density lesion in both of her lungs (Fig. 1a). Severe pneumonia was the diagnosis, and antibiotics (cefoperazone and shubatan) were prescribed. However, her condition deteriorated. For further diagnosis and treatment, she was referred to our department. On admission, rales and wheezes could be heard in both her lungs while she was in a sitting position during a physical examination. Laboratory and other examinations were conducted. Blood routine results were as follows: leukocyte, 14.8 × 10^9^/L; neutrophil percentage, 84.3%; lymphocyte percentage, 11.3%; sedimentation, 15 mm/hour; lactate dehydrogenase (LDH), 836 U/L; albumin, 28.6 g/L; and negative autoantibody spectrum, negative rheumatoid factor (RF), antistreptolysin O (ASO), and C-reactive protein (CRP); and human chorionic gonadotropin (hCG), 4016 U/L. A sputum sample culture was positive for the growth of *Klebsiella pneumoniae* bacteria. Bronchoscopy revealed the absence of bleeding and other abnormalities. A transbronchial lung biopsy (TBLB) was performed in the upper lobe of her right lung, and gestational choriocarcinoma was the diagnosis (Fig. 1b). On the third day of admission, her breathlessness increased. On the fifth day of admission, she had sudden massive fatal hemoptysis because of respiratory failure, and tracheal intubation and mechanical ventilation were performed. However, intervention therapy, such as interventional radiology or bronchoscopy, was not performed to locate the site of bleeding. She died on the fifth day of admission.

### Case 2

**Fig. 2:**
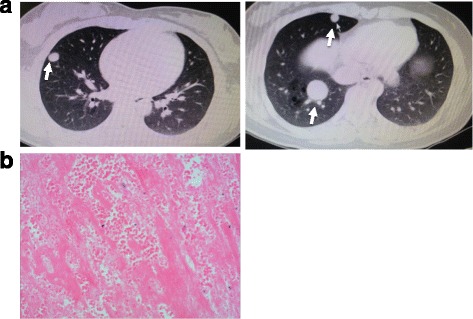
**a** Chest computed tomography shows multiple clear boundary nodules (*arrows*) with different sizes in the bilateral lung. **b** Lung tissue specimen shows necrotic tissue (hematoxylin and eosin × 200)

A 23-year-old Chinese woman of Han nationality complained about coughing blood for 7 days. One week before admission, she had a cold and coughed with blood. Fever, chest tightness and pain, and dyspnea were not reported. Chest CT images revealed multiple nodules of different sizes in both lungs, which could be diagnosed as lung metastasis (Fig. 2a). For further diagnosis and treatment, she was referred to our department. Since the onset of symptoms, she had normal mental status, diet, sleep, and defecation, and her body weight did not change significantly. She had a history of a mole and she underwent curettage 2 months prior to her admission. A physical examination on admission showed that both her lungs were clear, and rales and pleural friction sound were not heard. Her blood hCG was 7500 U/L. She underwent CT-guided percutaneous lung puncture; lung tissue pathology showed necrotic tissue (Fig. 2b). She was diagnosed as having choriocarcinoma.

### Case 3

**Fig. 3:**
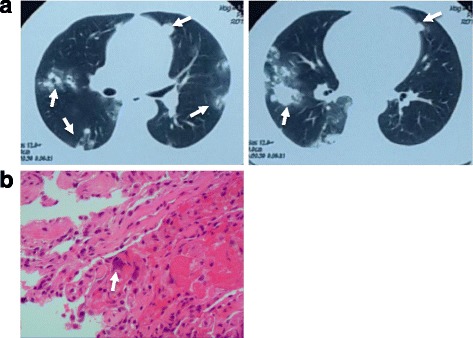
**a** Chest computed tomography shows multiple high-density nodules and patchy infiltrates with a halo sign (*arrows*) in both lungs. **b** Several atypical cells (*arrow*) are scattered in the lung tissue and bronchial mucosa squamous epithelial hyperplasia (hematoxylin and eosin × 200)

A 46-year-old Chinese woman of Han nationality was admitted for 5 days because of hemoptysis. She has a son and a daughter, and had had an abortion four times. She had menopause and was positive for a pregnancy test 60 days prior to admission. Five days prior to her admission, she had hemoptysis without obvious causes; the amount of blood was approximately 100 ml per day. She did not have fever, chest pain, or dyspnea. Four days before her admission she had a chest CT scan, and the images showed multiple high-density nodules with a halo sign in both lungs (Fig. 3a). Since the onset of the symptoms, she had normal mental status, diet, sleep, and defecation, and her body weight did not change significantly. A physical examination on admission showed that both her lungs were clear, and rales and pleural friction sound were not heard. Laboratory tests showed an hCG level of 207,900 U/L in her blood. Moreover, bronchoscopy revealed the absence of abnormalities and bleeding. A TBLB was performed in the anterior basal segment of the lower lobe of her right lung. Lung tissue pathology was negative (Fig. 3b). She was diagnosed as having choriocarcinoma based on her hCG level and history.

## Discussion

Hemoptysis is a common symptom of respiratory disease. However, choriocarcinoma with pulmonary metastasis is not a common cause of hemoptysis. The primary manifestation of the three patients was hemoptysis with cough, with or without dyspnea. Menstruation was significant in making the correct diagnosis. The patient in the first case was at her 36th week of pregnancy. The second had a history of hydatidiform mole, and the third patient had a history of abortion. In general, choriocarcinoma is secondary to a mole and to abortion, ectopic pregnancy, and preterm pregnancy or term pregnancy. The incidence of choriocarcinoma in China is high, approximately one case per 2882 pregnancies [1]. In Europe and North America, approximately one case per 40,000 pregnancies is reported. In Southeast Asia and Japan, 9.2 cases per 40,000 pregnancies and 3.3 cases per 40,000 pregnancies are reported, respectively. The incidence of choriocarcinoma lung metastasis was 85.1% [2].

The most common metastatic sites were the lungs (80%), vagina (30%), brain (10%), and liver (10%), whereas lymphatic system metastasis was rare [3, 4]. The above-mentioned phenomenon is related to the biological characteristics of choriocarcinoma, which are uncontrolled trophoblast stem cells and abnormal hyperplasia. These characteristics cause the loss of the original villi structure, vascular damage owing to invasion capability, the continuous infiltration and dissolution of endometrial stromal cells, and myometrium invasion, which leads to early blood vessel invasion and hematogenous metastasis. Choriocarcinoma cancer embolus sheds in vein reflux to the right side of the heart and then into the pulmonary artery, which embolizes small pulmonary artery branches. Then, the choriocarcinoma cell proliferates and invades the blood vessel wall, destroys lung tissue, and causes pulmonary metastases when mixed with hematoma. Afterward, the choriocarcinoma cell invades through the small pulmonary vein, returns to the left side of the heart, and transfers through systemic artery to the brain, liver, and every organ of the body. The lung is often the first site of hematogenous metastasis, and other organs are rarely affected [5]. In pathology, the center of lung metastatic lesions often shows clots and necrotic tissue surrounded by two layers of malignant trophoblasts. The inner layer is a mononuclear trophoblastic cell layer. Slight nuclear atypia and atypical mitotic figures may be observed, which are irrelevant to the prognosis. The outer layer is the multinucleated syncytiotrophoblast layer, which characteristically lacks chorionic villi and chorionic gonadal hormone promotion, and is positive for hCG staining. Compressed by transmitted lesions, the lung tissue around the lesion often collapse, and hemorrhage, edema, and inflammatory cell infiltration can be observed. Immunohistochemistry is helpful for the differential diagnosis. In syncytiotrophoblast, hCG is strongly positive, and human placental lactogen prime is weakly positive [6]. Chorioepithelioma hCG, inhibin and human leukocyte antigen-G, and melanoma cell adhesion molecule (Mel-CAM) are often positive [7]. The first patient tested positive for hCG. The other two patients did not undergo the test. The lung tissue pathology of the second patient showed necrotic tissue and the lung biopsy of the third patient was negative; immunohistochemical tests are not necessary for the clinical determination of choriocarcinoma. These immunohistochemical tests may play an important role in the differential diagnosis of the disease.

If a woman of childbearing age has a history of choriocarcinoma or irregular vaginal bleeding, postpartum or owing to abortion, which is accompanied by an elevated hCG level in her blood and urine, then choriocarcinoma may be the diagnosis. Combined with appearance of metastasis as shown in lung CT, the clinical diagnosis of choriocarcinoma with lung metastasis could be made. A definitive diagnosis requires a pathological examination of a uterine specimen or metastasis after resection. Choriocarcinoma cells secrete hCG, which can help in the diagnosis, therapeutic evaluation, and follow up of patients with choriocarcinoma. Tests for hCG have considerably high sensitivity and specificity.

The most common sites for metastasis are in the lungs. In the early stage of choriocarcinoma with lung metastasis, patients usually have no obvious clinical manifestations. Hemoptysis, chest distress, and chest pain may present in severe cases with multiple and larger metastases, and even hemothorax or severe respiratory failure can occur in the most serious cases. Currently, patients diagnosed with choriocarcinoma are advised to undergo a chest CT scan to check for lung metastasis, and this approach can often help in the early detection of lung metastasis, particularly in asymptomatic patients. For patients with hemoptysis as the initial symptom, if there are high risk factors, physicians should be aware of the possibility of a choriocarcinoma diagnosis.

The chest imaging manifestations of pulmonary metastatic choriocarcinoma vary. Metastatic lesions may be isolated or multiple. Several miliary lesions and solitary or multiple nodules or masses can be observed and expressed as flaky, patchy, or cotton-like lesions [8–14]. These signs can occur alone or at the same time and can transform into each other in the development of the disease. A cavity lesion, which shows a thick wall cavity, is occasionally observed [15]. Endobronchial metastasis is unusual. However, this type of metastasis is still reported [16].

The treatment for choriocarcinoma is chemotherapy, combined with surgery, radiotherapy, and interventional therapy. The low-risk patients who have low resistance for methotrexate (MTX) or actinomycin D (ActD) can be treated with a single therapy, whereas the high-risk patients need a multidrug therapy. Most of the lung metastases can be treated after chemotherapy. Only a small number of patients with lung lesions resistant to chemotherapy or recurrence need surgical treatment.

## Conclusions

Based on the diagnosis and treatment of the three patients, we can conclude that for women with a pregnancy history and hemoptysis, particularly in the presence of risk factors, such as abortion, hydatidiform mole, ectopic pregnancy, and age (greater than 35-years old), choriocarcinoma may be the possible diagnosis or at least the main differential diagnosis. Constant vigilance is necessary to improve diagnostic efficiency.

## Abbreviations

ActD: Actinomycin D
ASO: Antistreptolysin O
CRP: C-reactive protein
CT: Computed tomography
hCG: Human chorionic gonadotropin
LDH: Lactate dehydrogenase
Mel-CAM: Melanoma cell adhesion molecule
MTX: Methotrexate
RF: Rheumatoid factor
TBLB: Transbronchial lung biopsy
